# Risk Stratification of Pulmonary Thromboembolism using Brain Natriuretic Peptide and Troponin I; a Brief Report

**DOI:** 10.22037/aaem.v10i1.1453

**Published:** 2022-01-08

**Authors:** Mohsen Ebrahimi, Mohammad Mohsen Arab, Hamid Zamani Moghadam, Majid Jalal Yazdi, Esmail Rayat doost, Mahdi Foroughian

**Affiliations:** 1 Department of Emergency Medicine, Faculty of Medicine, Mashhad University of Medical Sciences, Mashhad, Iran.; 2 Department of Cardiology, Faculty of Medicine, Mashhad University of Medical Sciences, Mashhad, Iran.; 3 Department of Emergency Medicine, Jahrom University of Medical Sciences, Jahrom, Iran.

**Keywords:** Troponin, Pulmonary Embolism, Natriuretic Peptide, Brain, Computed Tomography Angiography

## Abstract

**Introduction:**

Pulmonary thromboembolism (PTE) is one of the most prevalent medical disorders, with a notable annual fatality rate. This study aimed to evaluated the accuracy of serum pro-BNP and troponin I levels in PTE diagnosis.

**Methods:**

This cross-sectional study was implemented on 267 patients with suspected PTE (sudden chest pain or sudden dyspnea) in Imam Reza Hospital in Mashhad, Iran. All patients underwent pulmonary computed tomography (CT) angiography (as the gold standard test) and their serum levels of troponin I and pro-BNP were measured. The screening performance characteristics of pro-BNP in detection of PTE cases were measured and reported using receiver operating characteristic (ROC) curve analysis.

**Results:**

Two-hundred-sixty-seven patients with a mean age of 67.7 ±11.5 years were evaluated (60.1% male). PTE was confirmed via CT angiography in 121 patients. The area under the ROC curve of troponin I and pro-BNP in detection of PTE was 0.501 ng/mL and 0.972 pg/mL, respectively. The sensitivity and specificity of proBNP at the best cut-off point (100 pg/ml) were 85.4% and 80.2%, respectively. The sensitivity and specificity of troponin I at the best cut-off point (0.005 ng/ml) were 65.5% and 42%, respectively.

**Conclusion:**

Due to the comparatively good sensitivity and specificity of proBNP in diagnosis of pulmonary thromboembolism, it can be employed as a diagnostic determinant in patients with suspected pulmonary thromboembolism along with other laboratory tests.

## 1. Introduction

Pulmonary thromboembolism (PTE) can range from being asymptomatic to extensive emboli with a high mortality rate. In the United States, 300,000 people die from PTE every year, and in China, PTE is much more common than it was 10 years ago (1). Smoking cigarette, malignancies, obesity, age, heredity, prolonged comorbidities, and surgical history are introduced as some of the risk factors of PTE (2). Most PTEs are associated with respiratory distress, chest pain, presyncope or syncope, and bloody sputum (3-5). Several biomarkers, including pro brain natriuretic peptides (pro-BNP) and cardiac troponins, have recently been considered for risk stratification of suspected PTE cases (6). Pro-BNP is mainly secreted from ventricles that are under the stress of altered hemodynamic status and congestive heart failure. Pro-BNP is a specific and sensitive indicator for evaluating ventricular function. It is also a suitable indicator for diagnosing heart failure in heart disease (7, 8). Kucher et al. showed that pro-BNP higher than 90 pg/ml could predict the risk of mortality as well as need for cardiopulmonary resuscitation and mechanical ventilation in PTE patients (9). In a meta-analysis by Lega et al., increased pro-BNP and troponin levels were directly related to worse prognosis in PTE patients (10). In addition, troponin I and T are known to increase in PTE and its levels correlated with mortality rate (11-15). 

According to the 2014 European Society of Cardiology (ESC) Guidelines on the diagnosis and management of acute pulmonary embolism, no studies have yet been performed to evaluate the appropriate cut-off point for BNP and troponin in diagnosis of PTE (16). Therefore, this study aimed to evaluated the accuracy of serum pro-BNP and troponin I levels in PTE diagnosis. 

## 2. Methods

***2.1 Study design and setting***

This cross-sectional study was performed on patients with suspected PTE, who referred to the emergency department of Imam Reza Hospital, Mashhad, Iran, from January 2017 to January 2018, to evaluate the diagnostic accuracy of serum troponin I and pro-BNP in detection of at-risk patients for PTE. Before enrolment of patients, the research process was explained and informed consent was obtained from them. Throughout the study, researchers adhered to the principles of the Helsinki Declaration and the confidentiality of patient information. All costs of the project were covered by the researchers and no additional costs were incurred by the patients. This study was approved by the ethics committee of Mashhad University of Medical Sciences under the ethical code IR.MUMS.REC.1395.

***2.2 Participants***

The study was performed based on the Standards for Reporting of Diagnostic Accuracy Studies (STARD) checklist. The sampling method was non-random and purposive convenience sampling was performed, including all patients who were eligible for study during the study period. Patients with sudden shortness of breath and chest pain and positive D-dimer levels were included. Not agreeing to participate in the study, pregnancy, renal failure, treatment with anticoagulants, myocardial infarction, need for intubation, myocarditis, massive embolism, hypertrophic cardiomyopathy, and negative D-dimer were considered as exclusion criteria.

***2.3 Procedure***

After history taking and comprehensive clinical examination of all patients, 5 cc of venous blood was drawn from the brachial vein to assess the serum level of troponin I, using Quantitative ELISA test kit (Diagnostic Automation Inc. the US), and N-Terminal pro-BNP, using CLIA Kit for Human (Cloud clone, US). The reference standard for PTE diagnosis was pulmonary CT angiography. 

***2.4 Data gathering***

Medical record review was performed by MF, ME, and MMA for assessment of eligibility. Medical records belonging to patients with suspected PTE, who had undergone CT angiography and whose pro-BNP and troponin levels were available, were considered for data extraction by HZM and MJY using a checklist of study variables. 

***2.5 Statistical analysis***

Patients' demographic and paraclinical information were coded and entered into SPSS software version 18. Chi-square test was used to analyze the qualitative variables and Fisher's exact test was used if necessary. Receiver operating characteristic (ROC) curve was used to determine the best cut-off point for pro-BNP and troponin I in diagnosis of PTE. P-value less than 0.05 was considered significant. Screening performance characteristics of pro-BNP and troponin I at the best cut-off points were calculated and reported.

## 3. Results

Two-hundred-sixty-seven patients with a mean age of 67.7 ±11.5 years were evaluated (60.1% male). PTE was confirmed via pulmonary CT angiography in 121 patients. Patients with and without PTE were similar regarding age (p = 0.775) and gender distribution (p = 0.28). Mean pro-BNP level was 282.6 ± 109.6 pg/ml in PTE cases and 49.6 ± 42.3 pg/ml in others (p = 0.001). Mean troponin I level in patients with and without PTE was 0.04 ± 0.09 and 0.04 ± 0.07 ng/ml, respectively (p = 0475).

***3.1 Screening performance characteristics***

**Figure 1:**
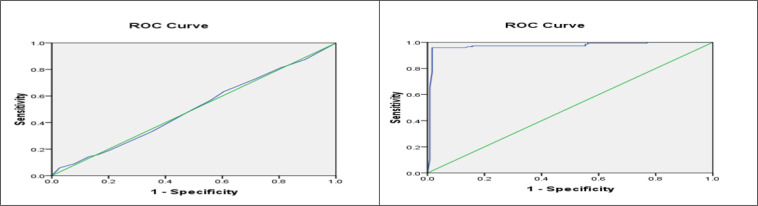
The area under the receiver operating characteristic (ROC) curve of troponin I (left) and pro-BNP (right) in detection of at-risk cases for pulmonary thromboembolism

The area under the ROC curve of troponin I and pro-BNP in detection of at-risk patients for PTE was 0.501 ng/mL and 0.972 pg/mL, respectively (figure 1). The best cut-off points of troponin I and pro-BNP in this regard were calculated to be 0.005 ng/ml and 100 pg/ml, respectively. The sensitivity and specificity of proBNP at the best cut-off point (100 pg/ml) were 85.4% and 80.2%, respectively. The sensitivity and specificity of troponin I at the best cut-off point (0.005 ng/ml) were 65.5% and 42%, respectively.

## 4. Discussion

In this study, we investigated the diagnostic accuracy of PRO-BNP and troponin in comparison to CT angiography as the gold standard to estimate the best fitting cut off levels of PRO-BNP and troponin in diagnosis of PTE. PRO-BNP had a sensitivity and specificity of 85.4% and 80.2% for PTE prediction in cut off level of 100 pg/ml. While in cut off level of 0.005 ng/ml, troponin I (Tpi-I) had low sensitivity and specificity (65.5% and 42%, respectively) in predicting the risk of PTE. 

In line with what we found in our study, in 2009 a meta-analysis by Lega et al. showed that elevated BNP and troponin levels were significantly associated with a worse prognosis in PTE patients (10). In Kucher’s study, the cut-off point of BNP in predicting the occurrence of adverse events in patients with PTE was 50 pictograms per ml with 85% sensitivity and 75% specificity (9). In another review study by Klok, subjects with acute pulmonary embolism experienced an increase in BNP levels in 51% of cases. Also, a direct relationship was found between increased BNP levels and right ventricular failure (P <0.001). In their study, patients with higher levels of BNP stayed in the hospital for a longer time and had more hospital complications, and also had higher mortality in 30-day follow-up (17). In our study, the diagnostic value of BNP and troponin in acute embolism was investigated and it was shown that an increase in BNP level to greater than 100 pg/ml had a sensitivity and specificity of 85% and 80%, respectively. But troponin had low specificity in diagnosis of acute embolism. It seems that pulmonary thromboembolism can increase BNP by acting on the right ventricle and causing some degree of ventricular failure, which can be used as a diagnostic marker in the acute phase, but the increase in troponin in the acute phase was not significant, as it takes more time for tissue ischemia to be developed.

## 5. Limitations

We know that history of previous medical conditions is very important in PTE development, so the generalization of our results to all patients with dyspnea and positive D-Dimer should be done with caution. 

## 6. Conclusion

Due to the relatively good sensitivity and specificity of PRO-BNP in diagnosis of pulmonary thromboembolism, it can be used as a diagnostic factor in patients with suspected pulmonary thromboembolism along with other laboratory tests.

## 7. Declarations

### 7.1 Acknowledgments

Thanks to Mashhad University of Medical Sciences for supporting and financing this article. Also, the clinical research development unit of Peymaniyeh Hospital of Jahrom city is appreciated and thanked for editing this article.

### 7.2 Authors’ contributions:

ME and ER conceptualized the study design. MF, ME, and MMA assessed cases for eligibility. HZM, MJY conducted data collection, ME and ER analyzed data. All authors contributed in manuscript drafting and revisions. 

### 7.3 Conflict of interest

The authors of this article did not mention any conflict of interest.

### 7.4 Funding and supports

Mashhad University of Medical Sciences has funded this study. 

## References

[1] Li H-L, Chan YC, Li N, Cui D, Cheng SW (2020). Prevalence and predictor of pulmonary embolism in a cohort of chinese patients with acute proximal deep vein thrombosis. Annals of vascular surgery..

[2] Gjonbrataj E, Kim JN, Gjonbrataj J, Jung HI, Kim HJ, Choi W-I (2017). Risk factors associated with provoked pulmonary embolism. The Korean journal of internal medicine..

[3] Miniati M, Prediletto R, Formichi B, Marini C, Di Ricco G, Tonelli L (1999). Accuracy of clinical assessment in the diagnosis of pulmonary embolism. American journal of respiratory and critical care medicine..

[4] Pollack CV, Schreiber D, Goldhaber SZ, Slattery D, Fanikos J, O'Neil BJ (2011). Clinical characteristics, management, and outcomes of patients diagnosed with acute pulmonary embolism in the emergency department: initial report of EMPEROR (Multicenter Emergency Medicine Pulmonary Embolism in the Real World Registry). Journal of the American College of Cardiology..

[5] Wells PS, Ginsberg JS, Anderson DR, Kearon C, Gent M, Turpie AG (1998). Use of a clinical model for safe management of patients with suspected pulmonary embolism. Annals of internal medicine..

[6] Daniels LB, Maisel AS (2007). Natriuretic peptides. Journal of the American college of cardiology..

[7] Hutchinson KR, Guggilam A, Cismowski MJ, Galantowicz ML, West TA, Stewart Jr JA (2011). Temporal pattern of left ventricular structural and functional remodeling following reversal of volume overload heart failure. Journal of applied physiology..

[8] Rondelet B, Dewachter C, Kerbaul F, Kang X, Fesler P, Brimioulle S (2012). Prolonged overcirculation-induced pulmonary arterial hypertension as a cause of right ventricular failure. European heart journal..

[9] Kucher N, Printzen G, Goldhaber SZ (2003). Prognostic role of brain natriuretic peptide in acute pulmonary embolism. Circulation..

[10] Lega J-C, Lacasse Y, Lakhal L, Provencher S (2009). Natriuretic peptides and troponins in pulmonary embolism: a meta-analysis. Thorax..

[11] Lankeit M, Friesen D, Aschoff J, Dellas C, Hasenfuß G, Katus H (2010). Highly sensitive troponin T assay in normotensive patients with acute pulmonary embolism. European heart journal..

[12] Giannitsis E, Katus HA (2005). Risk stratification in pulmonary embolism based on biomarkers and echocardiography. Circulation..

[13] Meyer T, Binder L, Hruska N, Luthe H, Buchwald AB (2000). Cardiac troponin I elevation in acute pulmonary embolism is associated with right ventricular dysfunction. Journal of the American College of Cardiology..

[14] Pruszczyk P, Bochowicz A, Torbicki A, Szulc M, Kurzyna M, Fijałkowska A (2003). Cardiac troponin T monitoring identifies high-risk group of normotensive patients with acute pulmonary embolism. Chest..

[15] Macrea M (2004). Cardiac troponin T monitoring and acute pulmonary embolism. Chest.

[16] Members ATF, Konstantinides SV, Torbicki A, Agnelli G, Danchin N, Fitzmaurice D (2014). 2014 ESC Guidelines on the diagnosis and management of acute pulmonary embolism: The Task Force for the Diagnosis and Management of Acute Pulmonary Embolism of the European Society of Cardiology (ESC) Endorsed by the European Respiratory Society (ERS). European heart journal..

[17] Klok FA, Mos IC, Huisman MV (2008). Brain-type natriuretic peptide levels in the prediction of adverse outcome in patients with pulmonary embolism: a systematic review and meta-analysis. American journal of respiratory and critical care medicine..

